# Views of patients with progressive illness and carers about the role of digital advance care planning systems to record and share information: A qualitative study

**DOI:** 10.1177/02692163241255511

**Published:** 2024-05-30

**Authors:** Jacqueline Birtwistle, Matthew J Allsop, Andy Bradshaw, Pablo Millares Martin, Katherine E Sleeman, Maureen Twiddy, Catherine J Evans

**Affiliations:** 1 Academic Unit of Palliative Care, Leeds Institute of Health Sciences, University of Leeds, Leeds, UK; 2 Cicely Saunders Institute of Palliative Care, Policy & Rehabilitation, King’s College London, London, UK; 3 Whitehall Surgery, Leeds, UK; 4 Institute of Clinical and Applied Health Research, Hull York Medical School, University of Hull, Hull, UK

**Keywords:** Patients, caregivers, digital technology, advance care planning, qualitative research, palliative care

## Abstract

**Background:**

Digital approaches are being explored internationally to support the elicitation, documentation and sharing of advance care planning information. However, the views and experiences of patients and carers are little understood, impeding the development and impact of digital approaches to strengthen palliative and end-of-life care.

**Aim:**

To explore perspectives of patients with progressive illness and their carers on digital approaches to advance care planning, anticipated impact from their use and expectations for their future development.

**Design:**

A qualitative study employing thematic framework analysis of data collected from focus groups and semi-structured interviews.

**Setting/participants:**

Purposive sample of 29 patients and 15 current or bereaved carers in London and West Yorkshire from hospice settings, non-governmental support and advocacy groups, and care home residents.

**Results:**

Four generated themes included: 1. ‘*Why haven’t you read what’s wrong with me?*’; uncertainty around professionals’ documenting, sharing and use of information; 2. The art of decision-making relies on the art of conversation; 3. The perceived value in having ‘*a say in matters*’: control and responsibility; 4. Enabling patient and carer control of their records: ‘*custodianship is key*’.

**Conclusions:**

Lived experiences of information sharing influenced trust and confidence in digital advance care planning systems. Despite scepticism about the extent that care can be delivered in line with their preferences, patients and carers acknowledge digital systems could facilitate care through contemporaneous and accurately documented wishes and preferences. There remains a need to determine how independent patient and public-facing advance care planning resources might be integrated with existing digital health record systems.

**What is already known about the topic?**

- Digital approaches are being explored internationally to support the elicitation, documentation and sharing of preferences determined through advance care planning activities.
- Despite moves towards enabling online patient access to digital advance care planning systems in the UK, there have been no reports of patient and carer involvement in their design, implementation or evaluation.

**What this paper adds**

- Varied approaches to advance care planning outside healthcare settings were identified, with mixed levels of interaction and interest in online resources to support the documentation of wishes and preferences for future care.
- Patients’ awareness of shortcomings in the accuracy and sharing of healthcare information about them led to scepticism and concerns about how well digital advance care planning systems could work.
- Participants welcomed the facility to view their own records specifically to check for accuracy and relevance, although they expressed caution about being able to edit their own records.

**Implications for practice, theory or policy**

- Patient and carer engagement elicited multiple requirements for digital advance care planning systems that can be used to guide future system development.
- Advance care planning outside healthcare settings may extend the reach of the process across diverse populations, but resulting plans need to be accommodated and available on existing electronic health record systems.
- Outcomes used to assess digital advance care planning need to reflect what matters to patients and their caregivers, including the presence of iterative discussions over time to ensure patients are fully informed about advance care planning decisions that are documented and shared.

## Background

Patients with long-term conditions or complex needs who are approaching the end of life should be offered the opportunity to take an active role in planning for their future health and care.^1^ Advance Care Planning is defined by the European Association for Palliative Care (EAPC) as a process that ‘enables individuals to identify their values, to reflect upon the meanings and consequences of serious illness scenarios, to define goals and preferences for future medical treatment and care, and to discuss these with family members and healthcare providers’.^2^ Patients’ preferences may be elicited iteratively over multiple discussions and should be available to healthcare providers so they can be accessed at the point of need.^3^ Numerous digital approaches are being explored internationally to support the elicitation, documentation and sharing of preferences determined through advance care planning.^4^ Digital approaches may capture varying elements of advance care planning information (i.e. advance statements of wishes and preferences, advance decisions to refuse treatment (e.g. cardiopulmonary resuscitation and antibiotics) and details of lasting power of attorney.^5^ Examples include patient-facing online resources to guide people through creating an advance care plan without direct input from a health professional.^6–10^ Such patient-facing platforms have been shown to increase engagement and documentation of advance care plans,^11^ but they typically require the download of a patient-completed paper-based form that needs to be taken to a health professional for adding to and storing in a digital health record. Paper copies of patient wishes and preferences lack utility if they cannot be accessed by their professional healthcare providers.^12^

Increasingly, digital systems are being used to support real-time documentation of advance care planning information by patients that can then be stored directly in their digital health records.^13–15^ Such systems are being used internationally in the USA,^16,17^ Australia^18^ and the UK.^19^ In the USA, online patient portals that enable direct editing of preferences and information (e.g. lasting power of attorney) in a patient’s medical record are viewed as acceptable to patients.^20^ However, most patient portals for palliative care are disease or site-specific,^20^ which may not reflect the capabilities of systems to share information across all settings involved in the care of a patient with palliative care needs. In contrast, Australia has implemented MyHealthRecord,^21^ a national online summary of a person’s health information that allows people to store, access and share their health and advance care planning information with their health professionals. There has been no exploration of patient and carer experiences and views relating to MyHealthRecord for advance care planning, although there is recognition of the need for future research to address a lack of trust and data privacy concerns among users.^22^

In the UK, digital systems are seen as a way to support the documentation, storage and sharing of advance care planning information across all settings involved in the delivery of palliative care.^3^ This is aligned with calls to integrate information technologies in a way that enhances patient experiences.^23^ In the UK, systems used for digital advance care planning are referred to as Electronic Palliative Care Coordination Systems (commonly abbreviated to EPaCCS).^13^ There has been variation in how EPaCCS are implemented in the UK.^24^ Whilst patients can have online access to their full primary care record,^25^ no system currently enables patient access to their advance care planning information within their digital health record.^24,26^ Despite moves towards enabling online patient access to advance care planning information, there are no reports of patient and carer involvement in the design, implementation or evaluation of EPaCCS.^13,15,27,28^ It is crucial that patients and families are involved to ensure future systems meet their needs. Our study aimed to explore patient and carer perceptions of a digital advance care planning system approach used widely across England (i.e. EPaCCS) that supports the digital documentation and sharing of care preferences.

## Methods

### Study design

A descriptive qualitative design employing thematic framework analysis was adopted. This study is part of a larger project exploring multiple stakeholder perspectives on digital approaches to advance care planning.^24,29^

### Inclusion/exclusion criteria

The target sample comprised patients and carers. Patients were eligible to participate aged 18 years or older with a life-limiting condition; carers or family members of patients were eligible aged 18 years or over and were currently caring for or had cared for a person with a life-limiting condition.

### Setting

The study was conducted in two regions in England: West Yorkshire and London (serving adult populations of 1,822,400 and 6,904,100 respectively). Both regions are known to have digital approaches to advance care planning embedded in services.

### Sampling

Participants were purposively sampled to ensure the representation of patients and carers with and without experience of using palliative care services.

### Recruitment

Participants were recruited from four hospices, two non-governmental support and advocacy groups, one care home and three Patient and Public Involvement and Engagement (PPIE) groups from acute hospitals. Eligibility and those responsible for recruitment varied by setting (Supplemental Appendix 1).

### Data collection

Data were collected between May 2022 and May 2023. Due to COVID-19 restrictions and preferences on travel and social distancing, all participants were offered the choice of an individual interview or a focus group. Current carers could attend as an individual participant, or as a participant accompanying the person they were caring for (in a focus group or dyad interview). Interviews and focus groups were conducted by JB, MA and AB. Development of the topic guide was informed by discussions with a patient and public involvement group for the project, independent of research participants (see Supplemental Appendix 2 Interview schedule). Informed consent was given by all participants. Interviews were digitally recorded. Data collection was conducted in person at a university, hospice, community or care home setting or participant’s home or via an Online platform (e.g. Zoom). Field notes were taken and used to document contextual information and aid the reflexive process. Data were pseudonymised to protect identity.

### Data analysis

**Figure 1.**
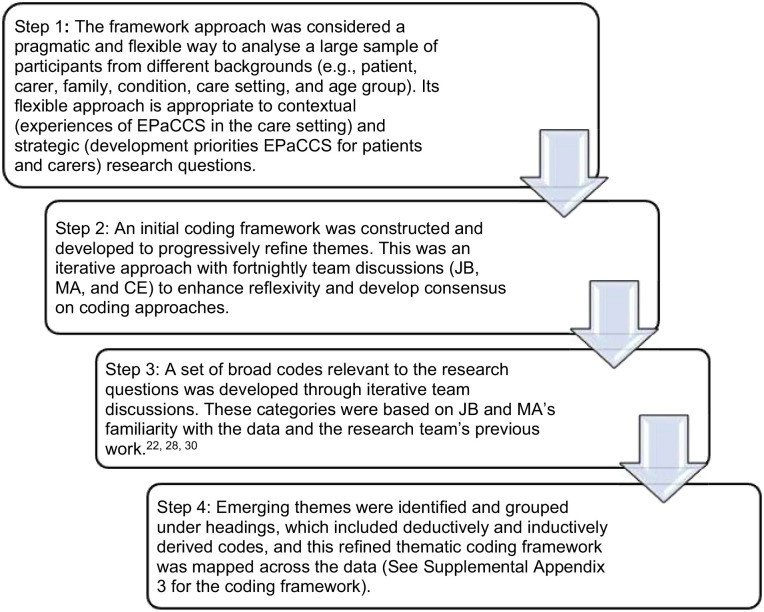
Overview of the data analysis approach using framework analysis.^4,13,15,24^

Interview data were audio-recorded, transcribed verbatim and analysed using thematic framework analysis.^30^ Data analysis using the framework approach was used, led by JB, working with CE and MA. All are experienced qualitative researchers in palliative care. The data analysis approach is outlined in Figure 1. Data analysis was conducted in tandem with data collection, and recruitment ceased when no relevant new codes were found in the data that added to the understanding of the research questions. The data management software NVivo V.12 was used to develop and refine a coding scheme and then index the interview transcripts. Microsoft Excel was used to chart the data. Consolidated Criteria for Reporting Qualitative Research (COREQ) guidelines have been followed.^31^

### Ethical approval

The Health Research Authority National Health Service, North of Scotland Research Ethics Committee reviewed and approved the study (21/NS/0046). All participants provided written informed consent.

### Patient and public involvement

The study was supported by patient and public involvement representatives who contributed to the design and development of the study and advised on recruitment processes, participant information and consent materials and the study topic guide.

## Results

Patients and carers from London (*n* = 15/*n* = 10, respectively) and West Yorkshire (*n* = 14/*n* = 5, respectively) participated in 8 focus groups and 15 individual or dyad interviews. Table 1 details participant characteristics and recruiting sites. Focus group size ranged from three to six participants and interview length varied for individual and dyad interviews (30–45 min long) and focus groups (60–120 min).

**Table 1.** Participant demographics and recruitment sites.

Demographic characteristics	Patient (*n* = 29)	Carer (*n* = 15)
Patient condition		
Cancer	11	–
Non-cancer	18	–
Known to specialist palliative care		
Receiving specialist palliative care	9	–
Not receiving specialist palliative care	20	–
Experience of participant having wishes and preferences documented		
On a digital advance care planning system	5	2
On a standalone digital system not linking to other health providers (e.g. within a care home)	4	–
Only using a paper-based advance care plan	5	6
Carer status		
Current	–	6
Bereaved	–	9
Region		
West Yorkshire	14	5
London	15	10
Age		
45–54	3	–
55–64	4	5
65–74	18	9
75+	4	1
Ethnic background		
White British	27	11
Asian	1	3
Black Caribbean	1	–
Mixed or multiple	–	1
Recruiting site or organisation		
Hospice	9	5
PPI group (Acute Hospital Trust^a^)	8	5
NGO^a^ (AGE UK, Compassion in Dying^b^)	8 (28)	5 (33)
Care home resident (palliative care needs identified by manager)	4 (14)	–

a Non-governmental organisation (support or advocacy group).

b Recruited via advertisement to mailing lists of respective organisations inviting expressions of interest from potential participants (patients with long-term or life-limiting conditions or carers of people with long-term or life-limiting conditions).

Four themes were generated: 1. ‘Why *haven’t you read what’s wrong with me?*’: Uncertainty around professionals’ documenting, sharing, and use of information; 2. The art of decision-making relies on the art of conversation; 3. The perceived value in having ‘*a say in matters*’: control and responsibility; 4. Enabling patient and carer control of their records: ‘*custodianship is key*’.

### ‘*Why haven’t you read what’s wrong with me?*’: Uncertainty around professionals’ documenting and sharing and use of information

Patients and carers rarely reported experience of documenting information about their preferences for future care using a digital approach. Of those with partial or complete digital records, none reported a situation where a health professional had cause to review their information. Some patients, and carers, had independently completed advance care planning information using an online resource (e.g. Macmillan Preferred Priorities for Care,^6^ Compassion in Dying Advance Statement^32^ and My Wishes,^9^) that could be saved as a printable copy. Few had transferred or replicated this on a digital system operating within a healthcare setting.

Participants expressed concerns that incorrect information could be shared widely using digital systems. They also considered whether their documented information was detailed enough to allow a future health professional to act upon their preferences in the way they had specified:

*it’s not ‘I want to be cared at home that means I don’t want any medical care anywhere else’, it depends on the situation and what the chances are, what the prognosis is and I’m not sure that that is recorded in any detail* ***ID: P08 Patient [Cancer]***

Some patients and carers discussed having elements of an advance care plan documented, including self-recorded (such as a Living Will or Lasting Power of Attorney) or with a health professional (such as an Advance Directive). Following the death of a loved one, bereaved carers reported being motivated to record their own wishes and preferences. If a paper-based advance care plan was completed with a health professional, there was an expectation for the person or family to share the information with other health services, such as the GP to upload it onto a patient’s digital record. The effort required to achieve this outweighed any benefits of sharing them:

(*Hospice) provided me and Mum and Dad with a My Future Wishes booklet and she said, ‘the best way to get this into the GP’s record is to scan it and send it to the GP surgery and they’ll attach it to the GP record.’ Well yeah, that’s a bit too much work when you’re faced with what we were faced with. We didn’t end up doing that in the end because actually, it would have been a lot of effort for little return. . . we basically carried it around in a little folder with us*. ***ID: P44 Carer [Bereaved]***

Patient participants often reported that they received care from many health professionals in different community and secondary care services, including ambulance services. Their experiences of information sharing between different health services informed their perceptions of the transfer of advance care planning information. Participants reported experiencing delays in their health information being documented in one service and then accessed by another. It was presumed this would occur for wishes and preferences documented on digital record systems. Participants were aware that scanned information, in particular, took time to upload to digital record systems and that some information could not be shared between systems at all:

*she [physiotherapist] was asking me what medication I was taking and I couldn’t remember the name of something, she said, ‘hang on I’ll look it up for you but I’ve just got to change systems ‘cos the GPs use a different system from me,’ and I thought, ‘well my GP’s only just up the road for goodness sake, why are they using two different systems, why can’t they access their systems’.* ***ID: P16 Patient [Non-cancer]***

Patients who completed online advance care plans independently were often hesitant to share their plan with a health service for it to be recorded in a digitally shared record. Their reluctance was often related to presumed difficulties accessing GP appointments and being from ‘*an oldish population, many of us were brought up never to bother the GP*’. *(ID: P39 Patient [Non-cancer])*. In some cases, participants did not trust that health professionals would read advance care planning information, even if it was available. Instances, where participants knew the health professional had not accessed their information, contributed to their lack of confidence:

*You think ‘why didn’t you read my records?’ you know what I mean, ‘why haven’t you read what’s wrong with me?’ It’s on the screen right in front of them*. ***ID: P14 Patient [Cancer]***

Participants were aware of the limitations of digital systems to support care in line with documented preferences at the point of emergency. They expressed concern that resuscitation would be attempted, regardless of expressed wishes in the case of a patient being unable to give their details. Suggestions for drawing attention to prior documentation of preferences in their digital records included having tattoos or bracelets, or carrying a paper version:

*You know it’s like, you know they ask you your name, you can’t respond, what are they going to do? They’re going to try and get you going again in a situation, so it’s very difficult to have a system that works for those emergency situations. Now personally [I] think that a digital system wouldn’t even work because how would the emergency staff know who to look for? You know, you’d have to have some form of identification - that they could check who you are like a tattoo of a number on your arm like an ID number almost. Yeah, I just, I’ve thought about it quite a lot and the easiest logical solution would be an ID bracelet* ***ID: P31 Patient [Non-cancer]***

### The art of decision-making relies on the art of conversation

Participants recognised that quality discussions were necessary at the time of documentation to ensure professionals interpreted wishes and preferences as the patient intended. The key characteristics of the health professional supporting the person to make and document informed choices were based on time to have a quality discussion with the patient alongside their clinical knowledge:

. . .*it needs, in an ideal world, a specially trained person to do this who was not burdened with other tasks, who was psychologically trained to know how to initiate those conversations but also was clinically trained to know all the different scenarios that you might be faced with to say, ‘Well look, what if this happened, what would you want here or there?’ You know, not just ‘OK, I’ve got a form quick tell me do you want to die in a hospital or at home?’* ***ID: P04 Carer [Bereaved]***

Participants also suggested social care practitioners who could support discussion and documentation of preferences. For example, home care workers’ expertise, commitment to delivering day-to-day care and knowledge of the patient were identified as having unrecognised potential for involvement in reviewing and updating digitally recorded preferences and supporting patients with decision-making:

*Even if you felt that the initial palliative care consultation ought to be done by a palliative care consultant, the ability to annotate and move that plan along with comments and things that the patient wants to change or anything like that ought to be accessible to other care providers. If they [home care workers] are in front of the patient then they’re capable of supporting a terminal patient, you can’t have it both ways, you can’t say, ‘they’re alright to give care but they’re not alright to record information.’ That sounds a bit weird to me. The 85% of the care that a seriously ill person gets is from a completely utterly unqualified person . . . They’re giving intimate care so of course they’re going to have a conversation as well*. ***ID: P44 Carer [Bereaved]***

Participants were undecided about the right time to document a digitally shared plan, even though they had started discussing their wishes and preferences with family members. What was important was to be able to consider their choices over time, and have the flexibility to change documented preferences when they identify a need:

*. . .we’ve [partner and patient] had the conversations you know, ‘You’re going into surgery, what if you don’t come back?’ They’ve had to be had between myself and my partner so we’ve written a will, and he knows down to the nth degree of what I want if I should ever be taken quickly but actually planning it and putting it down on paper, no we haven’t done that or planned it you know electronically . . . I know that plans can be put in place but just the in-depthness and how detailed the plans can be I don’t really understand that if I’m honest. . .. But that’s because I’m probably not at the stage where I feel the need to do that yet. But it is something I have thought about.* ***ID: P25 Patient [Cancer]***

Participants expressed the importance of distinguishing between preferred responses to potentially different scenarios. However, they felt it was difficult to predict the impact of interventions such as cardio-pulmonary resuscitation on their quality of life and valued the opportunity to hear about likely outcomes to understand the implications of potential treatments:

*People have asked me about DNR [Do Not Resuscitate] and things like that. . . I’ve told them what I think and everything and if they don’t think resuscitation is actually going to give me a, you know, bring me back properly and give me a quality of life then I don’t want it. On the other hand, if they do think that resuscitating me will give me some kind of quality of life then I do want it. I don’t know, I’m undecided*. ***ID: P12 Patient [Non-cancer]***

There was recognition of the need for support from a professional with time to help patients think through alternative scenarios without overestimating the patient or the carers’ capacity to understand the implications of different choices:

*I mean this is a general phrase, ‘making someone comfortable,’ what does that mean, you know, that I mean even I, who have spent such a long time advocating for my mum when she was actually dying and people were asking me, ‘what do you want?’ and they’re trying to be tactful about it. And I’m going, ‘well, obviously I want you to make her comfortable.’ But then I don’t know what they mean by that. You know, ‘what are you actually going to give her to make her comfortable, what will be the outcome of that?’ . . . even before we can discuss who gets access to the records, I think you have to go right back to basics and say people really need to understand what they’re being asked to decide and then they might want to have that record there.* ***ID: P04 Carer [Bereaved]***

Central to the concept of person-centred care was the importance of involving the patient in the process of documentation and subsequent sharing and recording of advance care planning information. Not involving the patient meant they felt disconnected from the process with little understanding of what was being recorded with no opportunity to confirm the health professionals’ understanding of their information. This led to anxieties about the quality of information and concerns about where it was shared. They considered potential solutions that ‘*would be very easy to do*’ *(ID: P44 Carer [Bereaved])* such as using dual screens or simply showing the patient what had been recorded.

*There is no concept in my mind of patient-centred care if patients don’t get to be part of the information sharing . . . in the vast majority of those instances is somebody’s asking you questions and then concentrating on a computer screen and tapping in information which you’ve got no idea what they’re writing, it feels like you’re in a police interview really. . . . You’d no idea what they were writing, there was no concept of turning round the screen and looking at that information and then to make it worse, you don’t really get that information it then goes off into the ether.* ***ID: P44 Carer [Bereaved]***

### The perceived value in having ‘a say in matters’: Control and responsibility

Some participants expressed that they felt reassured by documenting their wishes and that it gave peace of mind for them, and their families, to have discussed and made plans for their future care.

*it’s only with the recent diagnosis that I realise I do need to put something in place because we did have a conversation recently because I do have this life-limiting condition and it affects my quality of life . . . I didn’t realise how nervous he [husband] was about should anything happen, you know should I have a heart attack or a stroke, what would he do. So I now feel the need to put something in place*. ***ID: P42 Patient [Non-cancer]***

Participants valued the autonomy that making decisions and recording their choices allowed them to have ‘*a say in matters*’ *(ID: P07 Patient [Non-cancer])* rather than health professionals and family members making decisions on their behalf. It was also considered a way of regaining a feeling of control that was lost due to their diagnosis and treatment:

*I can’t control my cancer so I am constantly looking for ways of saying’, ‘well I can control that. I can’t control the tumour but yes I can control that,’ and it makes me feel stronger and it’s better for morale.* ***ID: P10 Patient [Cancer]***

Despite increasing a sense of autonomy, it could also raise feelings of doubt in their own choices and that health professionals would make better treatment decisions:

*I suddenly realised that I have now taken responsibility for aspects of my care which previously I have been perfectly happy for the professionals. So I was a little concerned that there might be things that I said on those forms that maybe, certainly as time goes on, but that could be in a certain light, in a certain day I would no longer approve of, it could be something which I, yesterday I thought was exactly what I wanted but you know, tomorrow, there’s something about the circumstances where if I’d left it to the professionals . . . that they would make the decision which is better than the one I made in advance without seeing the whole situation.* ***ID: P36 Patient [Non-cancer]***

Participants were hopeful that at the point of need, health professionals would act upon the preferences documented by the patient and reduce the potential for family members to override patient decisions about their care:

*I needed to do that [document wishes] to ensure that her wishes and her desires were captured and then also to be able to communicate to my siblings. So, I did that for sort of self-satisfaction and dignity and respectfully but actually it’s become much more important now because if she is losing her cognitive senses then you know at least I now know what her wishes were.* ***ID: P16 Carer [Current]***

Participants without nearby family envisaged that the presence of digitally available preferences freed them from asking friends to advocate for their care who might ‘*say ‘yes’ because they don’t know how to say ‘no’ to me or they make a mess of it’ (ID: P17 Patient [Non-cancer])* or were also at the point of making similar decisions for themselves or supporting others.

### Enabling patient and carer control of their records: ‘Custodianship is key’

Access to their digitally recorded advance care plan could enable participants to check that information aligns with their intended preferences. It could also provide an opportunity to alert health professionals to revisit and amend information that is inaccurate, incomplete or out of date:

*if everyone else is able to access this and I can’t, is there something incorrect in there . . . that really frightens me to think that you are at the mercy of somebody typing a lot of data in* ***ID: P04 Carer [Bereaved]***

*Custodianship of information is really key here because we talk a lot in the NHS about patient-centred care and patients being at the centre of the care but when they don’t own their care plan and often don’t have access to their care plan, then they can’t be the owner* ***ID: P44 Carer [Bereaved]***

Both patients and carers welcomed carer or family access to view the record. It was important that patients could approve who sees it and some welcomed an alert to tell them who had accessed their records. Family access could facilitate inclusion in care and planning by communicating challenging information to different family members:

*it kind of feels a little bit as if you’re doing something together. Yes, absolutely yeah because they can share it. They can share it with you without actually having to have really heartfelt conversations ‘cos they’re hard you know. They could access it and process it . . . ‘cos then everybody can look at it and everybody can keep abreast of what’s going on as well’ ‘cos one of the hardest things I find is you tell the same story. So I go to the hospital and then I discuss it with [husband], I discuss it with the children, I discuss it with my brother’*, ***ID: P25 Patient [Cancer]***

Most participants did not want to edit their information independently, and valued the opportunity to review the record with a professional:

*I don’t think I would be updating it like every three seconds if you see what I mean, it wouldn’t be that, hopefully that often so and therefore I wouldn’t be having a conversation either. But I suppose I would like to have the conversation; I like the personal interaction with my doctor for example not just typing something out in my electronic record if you see what I mean. If there’s a change, a significant change you know.* ***ID: P06 Patient [Cancer]***

Concerns about patients’ editing rights were often related to the potential risk of coercion to record information suggested by people known to the patient. Some patient participants indicated they did not want family members to see sensitive information, such as sexual or mental health entries:

*whether or not people wanted their mental health records put on there or their sort of sexual health records put on there for people to see and people like carers, then you’d get you know, you’ve got this, you started up with a whole can of worms.* ***ID: P17 Patient [Non-cancer]***

Patient participants wanted to have control of which care settings could view their information, and to be able to review and change their decision about setting access:

*I have asked my GP not to share my records outside of the GP practice without my permission and that is because, with the Leeds Care records which is something which has been created within Leeds as I understand it, the people who can access it are not just medical care workers they are also care assistants in care homes. . . . And going back and saying actually I think I want to change my mind about that and how do you go about doing it? So that’s part of the sharing and the access, you can make initial choices but I might end up exactly the same as [P42] saying I’d like everybody to have access, how do I go about changing my mind and saying ‘please make it available to anybody who has anything to do with my end-of-life care’? And I may well want that in the end* ***ID: P43 Patient [Non-cancer]***

While participants described older patients who were capable of accessing their information (e.g. quote from P44 [Carer]), participants commonly raised the issue of digital exclusion inhibiting access to information for patients unable to use digital technology or access the internet, and older age was widely cited as a barrier (e.g. quote from P25 [Patient]):

*I don’t think that the age, that the inaccessibility of technology is as big as people think. So, my dad’s 84 now and he talks about being completely technophobic really, but he still has a smartphone and he still has an app that he uses, he still has an iPad that he uses every night to read the news and to play games and things on. My mum was absolutely capable of that she did all of her own banking online, . . . she had the NHS app and she had managed to download COVID certificates on her own in order to travel only 2 months before she got her diagnosis, and she was 81.* ***ID: P44 Carer [Bereaved]***

*I’m a different generation to a lot of the patients. A lot of patients are elderly aren’t they so would they want that? Probably not because they wouldn’t have the technology you know like using my mum for example, my mum doesn’t even have the internet, she doesn’t have a computer.* ***ID: P25 Patient [Cancer]***

Digital systems should be easy to use and access without compromising the security of records. Furthermore, the facility to print their advance care planning was important. Participants valued being able to show this to health professionals who could not access the respective digital system at the point of need.

*I think it would be helpful as a patient to have like a hard record on paper. At least I’d know what was there even if I changed it, I could add my own you know or something, I’m very nervous about everything being I don’t know, somewhere electronic.* ***ID: P08 Patient [Cancer]***

Participants discussed the type of information that would be useful to record. As well as being able to express treatment preferences and where they would like to be cared for, participants specifically focussed on the importance of documenting more holistic information about themselves Participants noted that health professional conversations were often focused on resuscitation needs and omitted what was most important to enable the person to have care needs met.

*They did need a DNACPR in place ‘cos that was appropriate, I understand that but the better questions to ask my mum would have been ‘what did dying look like for her?’ You know, ‘who was around her, who was with her, where was she?’ Things like she really enjoyed sweet food and she wanted her family with her as much as possible to be able to stay with her, that was really important to her, she didn’t like to be in a room where there were lots of other people, she found that a little bit stressful.* ***ID: P44 Carer [Bereaved]***

Participants envisaged the type of personal information that would enable them to receive better care in different situations and care settings, including emergency scenes of care. Participants also outlined multiple types of data that they would like to store in digital records to guide care that supports patients’ self-respect and dignity. These included personal information on adaptations required for disability or limits in functioning, how patients may want to be treated in terms of social interaction (e.g. ‘*these records need to have a more rounded view of what makes this person tick . . . in hospital, I had to try and chat to as many people as I could. . . then there were people in the hospital who did not want to talk to anyone*’ *(ID: P22 Patient [Cancer])*, and comfort (*e.g. ‘I suffer really badly with my feet through my chemotherapy . . .something that I need to do every day is that my feet are creamed 4 times a’ (ID: P25 Patient [Cancer])*; ‘. . .*in terms of pain or hydration or anything else . . . it’s also about specifying what you’d like for mouth care*. . .’ *(ID: P42 Patient [Non-cancer])*, what patients enjoy doing, and adaptations required for specialist diets relating to food intolerances.

## Discussion

### Main findings

Patients’ engagement with discussions and subsequent documentation of advance care planning wishes and preferences was varied. Some participants actively pursued this via online resources while others were clear that this was not wanted or did not know it could be put in place. Lived experiences involving digital health records in any aspect of prior care influenced participants’ views on how using digital advance care planning would support their future care. Almost all participants described situations where health information had not been shared between services when expected, and many recounted instances of incorrect information about them being recorded. This led to concerns around the accuracy of advance care planning information, patients not knowing what information, if any, was already held about them, and who could see it. There was also recognition of the need for a quality discussion to support advance care planning decision making but it was not always clear which health professional could be approached to support this discussion. Patients were often hesitant to approach a general practitioner for this type of discussion, due to perceived difficulty in obtaining a face-to-face appointment.

### What this study adds

Existing and emerging systems designed for advance care planning information documentation and sharing have lacked patient and carer engagement to guide their development and content. In Table 2, we present a list of considerations for the future development of digital advance care planning systems that facilitate patient access to their records.

**Table 2.** Patient and caregiver-focussed considerations derived from the study findings to inform the future development of digital advance care planning systems.

	Key findings	Considerations for digital advance care planning system design
Preparation and documentation of advance care planning preferences	Online patient information is being accessed and used by people to engage in or access information on multiple elements of advance care planning. *[Theme 1]* Participants recognised that discussions with health professionals could be inadequate and rushed. This led to concerns that shared wishes and preferences were not interpreted and documented as the patient intended. *[Theme 2]*	Health professionals may be able to draw on existing patient-facing resources to support preparation and readiness for advance care planning discussions. This may include information relating to the role and function of digital advance care planning approaches. However, there is variation in the quality of existing content on patient-facing resources.^34,35^ *[Theme 2]*
	Multiple, publicly available digital platforms are being used by people to support them to independently create a digital record of their wishes and preferences for care. *[Theme 1]*	There is duplication across publicly available digital advance care planning websites, alongside needs to improve their accessibility and languages across diverse populations, cultures and minority groups.^33,34^ *[Theme 1]*
	Community-based advocacy and support groups are working with clients to promote the uptake and use of advance care planning support and documentation. *[Theme 1]*	Community-based support spaces including businesses, legal organisations, disease or illness groups, funeral homes, homeless shelters, carer associations, seniors’ centres, primary care networks, unions, financial organisations and faith-based groups are novel settings where diverse populations could be supported to participate in advance care planning.^35–37^ There is scope to explore the involvement of existing community-based support groups to raise awareness of and facilitate discussions and processes surrounding digital advance care planning. *[Theme 1]*
	There are few processes in place to accommodate the transfer of information documented using online advance care planning resources outside of consultations in healthcare settings. *[Theme 1]*	Informal approaches to advance care planning, such as community-based support spaces, are providing routes to support engagement, particularly from underserved groups.^35,38^ The design of digital advance care planning systems may need to accommodate documentation generated outside healthcare settings that, whilst not aligning with fields in existing structured documentation, may still contain information relevant to a person’s preferences for care and management. *[Theme 1]*
Content to capture advance care planning wishes and preferences	Reflecting the holistic account of key patient characteristics, needs and preferences is best reflected through free text information. *[Theme 3]*	Published standards^39,40^ suggest that medical, social and psychological information, with an emphasis on free-text information, may provide a holistic account of key patient characteristics, needs and preferences. This information may have value and guide person-centred care across settings, including the accurate documentation of information within electronic medical records to inform decision-making and support the continuation of normality and self-identity.^41^ The functionality of digital advance care planning systems may need to include ways of presenting unstructured fields to accompany structured documentation around, for example, preferred places of care and death. [Theme 3]
Access to and viewing of advance care planning preferences	There is a desire for people to have access to their own records to address concerns relating to the accuracy and relevance of stored information. *[Theme 4]*	Digital advance care planning systems need to explore modes for enabling patient access to review information stored in their records. This could be, for example, via patient portals linked to digital health record systems, or through approaches such as mobile phone applications. *[Theme 4]*
	Patients and carers have reservations over being able to edit any documented advance care planning wishes and preferences. *[Theme 4]*	Digital advance care planning systems may consider developing a review function that would enable patients (and potential family members and carers) to suggest alterations to standardised or clinically coded advance care planning information that could be reviewed and approved by a health professional. However, systems could simultaneously provide more flexibility for editing free-text information relating to their characteristics, needs and preferences. *[Theme 4]*
	Patients would like the option of having viewing access for patient-designated family members or informal carers. *[Theme 4]*	Digital advance care planning systems may consider developing functions that would enable access to view documented wishes and preferences of a patient. This may provide support for families advocating for a patient. *[Theme 4]*
	Patients and nominated others want easy access to digital advance care planning that does not comprise security of their data. *[Theme 4]*	Irrespective of the user, digital advance care planning systems should ensure accessing, viewing and editing details relating to documentation is simple and aligned with common processes across online resources such as using social credentials (i.e. a single sign-on using existing information from a social networking service) or a one-time passcode process. *[Theme 4]*

The impact of digital advance care planning important to participants in our study includes documentation of quality-of-life aspects such as personal autonomy and emotional, social and spiritual factors. Commonly digital advance care planning systems are evaluated using outcomes including the location where a person is cared for and dies^4^; metrics that do not indicate care quality of the experience of the patient.^42^ This mismatch is echoed in the UK where the intended impacts of digital advance care planning approaches are aligned with national policy goals (e.g. early identification and recording of people approaching end of life and reducing avoidable hospital admissions^13,24^). Our findings can inform the refinement of outcomes for assessing digital advance care planning system use to include, for example, the presence of iterative discussions over time that are needed to ensure patients are fully informed about the decisions that are documented and shared. Furthermore, health professionals who support patients in documenting needs and preferences should be competent in both presenting information to support choices as well as instilling confidence that choices will be accessed and used to inform their care at the point of need. Health professionals should endeavour to be transparent about unknowns in terms of which services can access the record and what can be achieved in adherence to preferences. Evidence on both the limited availability of advance care planning information and its transferability between services suggests that health professionals could play a greater role in ensuring patients are informed about the storage of their information.^28,43^

This study arises alongside increasing medical information sharing with patients and reflections on the optimal amount and types of information to be shared.^44^ Where digital advance care planning systems incorporate patient and carer access, mechanisms should be in place that permit information to be viewed and edited by only patient-authorised others. Such approaches are being developed in the US, with patient portals that enable caregiver engagement with and use of patient portals in palliative oncology.^45^ These are deemed acceptable to patients and caregivers and perceived by clinicians to benefit clinical care.^46^

### Strengths and weaknesses

This study is the first that we know of to explore patient and carer experiences and views of digital approaches to advance care planning. A major strength was the involvement of patients using different health services and support organisations and the inclusion of both current and bereaved carers able to report on experiences and views of digitally documented wishes. Limitations included recruitment taking place when access to hospice services was limited by protective measures related to COVID-19. This impacted capacity at sites and the running of focus groups. Ethnic diversity was limited with participants mainly identifying as White, with future research needed to explore the breadth of factors that may influence needs and preferences across diverse and underrepresented groups. The digital systems used for advance care planning are currently only accessed by health professionals, so few participants were able to report their experience of digital documentation of advance care plans, and none could report on health professional access to review or use information recorded.

## Conclusion

If issues around accuracy, completeness, relevance and sharing of patient data in electronic health records continue to be encountered, then patients’ trust in the use of digital systems for end-of-life preferences will remain low. Despite reservations concerning security, accuracy, sharing and health professional access and adherence to preferences, there was an appetite for accessing digital resources and hope that systems for recording advance care planning needs and preferences could be beneficial to urgent and end-of-life care. Secure access balanced with minimal password burden to patients’ records may provide a route to check the accuracy and relevance of information. Future research is required to explore how existing online resources and advance care planning documentation generated with the support of community support groups can be accommodated by existing digital advance care planning systems.

## Supplemental Material

sj-pdf-1-pmj-10.1177_02692163241255511 – Supplemental material for Views of patients with progressive illness and carers about the role of digital advance care planning systems to record and share information: A qualitative study Supplemental material, sj-pdf-1-pmj-10.1177_02692163241255511 for Views of patients with progressive illness and carers about the role of digital advance care planning systems to record and share information: A qualitative study by Jacqueline Birtwistle, Matthew J Allsop, Andy Bradshaw, Pablo Millares Martin, Katherine E Sleeman, Maureen Twiddy and Catherine J Evans in Palliative Medicine
