# Supplementary material for: Views of patients with progressive illness and carers about the role of digital advance care planning systems to record and share information: A qualitative study
Source: Palliat Med. 2024 May 30;38(7):711–24. doi: 10.1177/02692163241255511 (PMC11290031; doi:10.1177/02692163241255511)
Supplement: sj-pdf-1-pmj-10.1177_02692163241255511 – Supplemental material for Views of patients with progressive illness and carers about the role of digital advance care planning systems to record and share information: A qualitative study [file sj-pdf-1-pmj-10.1177_02692163241255511.pdf]

Supplementary Appendix 1: Overview of eligibility and approach to recruitment of participants by setting

Setting	Eligibility	Person responsible for approaching potential participants
Hospices	Patient participants: registered on a palliative care clinical nurse specialist or specialist doctor caseload or to attend a regular hospice-based activity group or complementary therapy.<br><br>Carer participants: either current carers of registered patients or were attending bereavement support groups.	Hospice research nurses and doctors identified eligible individuals and carried out repeated searches for new listings monthly.
Care homes	Care home residents identified as having a palliative diagnosis.	Care home manager.
PPIE and Advocacy groups	People with personal experience of a life-limiting condition, either as a patient or carer.	Advertised the study to members of the public registered with their networks.

Participants who self-reported experience, expressed an interest, and confirmed consent to be contacted were then contacted by a member of the research team.

Supplementary Appendix 2: Interview schedule and topic guide

This workshop topic guide will be a living document.<br>*The design is iterative to be flexible to the needs of the group and the discussion that arises during the workshop.*<br>*It should be adapted as considered appropriate for individual, dyad and online interviews.*

1. **Welcome to the group**
   - Thank you for agreeing to take part in this focus group.
   - As you know, we are talking to patients and caregivers about their understandings, views and experiences of the use of electronic patient records systems in end of life care.
   - With your permission I would like to record the workshop; all details will be confidential and names will be changed to prevent identification.
   - There are no right and wrong answers to the questions.
   - If you do not hear or understand a particular question, please ask for clarification.
   - You can choose not to answer a particular question, without needing to give a reason
   - Please respect each other's privacy and confidentiality and take care not to repeat information about the other people attending today
   - Do you have any questions or concerns?

2. **Consent**
   - Review the information sheet and opportunity to ask questions
   - Obtain written consent
   - Complete demographic forms
   - Complete confidentiality agreements

3. **Introductions to the group/interview**
   - Brief introduction of interviewer(s) and their role, including research interests.
   - Invite participants to introduce themselves to the group.

4. **Introduction to the project**
   - What advance care planning is
   - How digital patient records systems can support documentation of advance care plans
   - Examples of where it might work and where it might not work

Start audio-recorder here and <u>announce that we are now recording</u>

Topic guide

General Opening

- What experiences have you had with shared patient records?
- What experiences have you had with sharing information about wishes and preferences for care?
    - Can you remember who told you about them and what you thought at the time?

Discussion 1: Patient and care priorities for sharing wishes and preferences for care

- Content of the record – what information should it include? How would this benefit you?
- Inclusivity – how can digital records for documenting and sharing patient wishes and preferences accommodate different individual and cultural needs?
- Approach (as a digital versus/supporting paper solution)?

Discussion 2: Patient and care priorities for accessing and sharing

Access

- Who do you think should access these records (e.g., patients, carers, family, healthcare professionals)?
- What do you think different people should be permitted to access?
- How do you think different people might access it?
- What would need to be in place for access to be made easy (e.g., technologies)?

Documentation and sharing

- Which health professionals/others should initiate discussions and document your preferences?
- Which health professionals and services should these be shared between?
- How would you like these records to be shared and with whom?
- When and what? Repeated conversations/ review conversations?
- What about confidentiality and data security?

Discussion section 3: Overall reflections and changes

- Were you aware of these records?
- Have you spoken about them with healthcare professionals?
- Do you feel involved in their creation and sharing?
- Priorities – what needs doing now - what can wait – what will need time to be developed?
- How do you think sharing plans will help services to run better?
- How do you think you would benefit from their use?

- What issues or concerns might arise for you or health professionals?

Appendix 3: Coding framework

Research question	Initial Coding framework and brief description	Refined themes
**Context**<br>Interview or focus group	**Session information**<br>About the group or individual/dyad interview	
**Context**<br>Personal	**A. About me:**<br>About the person (e.g. condition, family, social), which services and health professionals are involved	Theme 2: *if a support with decision or documentation need*<br>Theme 4: *if a system need*
**RQ1**<br>What are experiences of EPaCCS use?	**B. Experience:**<br>a. Digital documentation & sharing of ACP information preferences<br>b. Other ways of documenting ACP information<br>c. Electronic documentation & sharing of health information<br>d. Issues encountered with planning, documenting or sharing ACP or health information<br>e. Benefits of using EPaCCS to document ACPs	Theme 1
**RQ2a**<br>What access, and ability to update, do patients want from their own EPaCCS record?	**C. Preferences for patient and carer access**<br>a. Level of access view/edit<br>b. Carer access<br>c. Benefits of own access<br>d. Concerns about own access	Theme 4
**RQ2b**<br>What are patients' and families' preferences on how EPaCCS is used?	**D. Preferences for sharing information (who, what, when):**<br>a. Introduce, think & discuss<br>b. Document, review & update<br>**c.** Share, access, view & action	Theme 2
**RQ2c**<br>What should be prioritised to record and share on an EPaCCS record?	E. **System priorities:**<br>a. Type of information to document<br>b. Content of my record<br>c. What systems need to do to support use	Theme 4
**RQ2d**<br>What are the priority outcomes or benefits for patients with an EPaCCS record and their caregivers?	**F. Perceived value and benefit:**<br>a. Patients<br>b. Carers<br>c. Health and care services	Theme 3
**RQ2e**<br>What does a system need to do to meet individual needs?	**G. Inclusivity**<br>a. Digital inclusion<br>b. Equity in having an advance care plan<br>**c.** Anything else?	Theme 2: *if a support with decision or documentation need*

		Theme 4: *if a system need*
**Miscellaneous**	Any aspects to generate further research ideas	
**Not relevant**		

Theme 1: Why *haven't you read what's wrong with me?*'; uncertainty around professionals' documenting, sharing and use of information
Theme 2: The art of decision-making relies on the art of conversation
Theme 3: The perceived value in having "*a say in matters*": control and responsibility
Theme 4: Enabling patient and carer control of their records: "*custodianship is key*"
